# Thromboembolic Complications in COVID-19 Patients Hospitalized in Italian Ordinary Wards: Data from the Multicenter Observational START-COVID Register

**DOI:** 10.1055/a-1878-6806

**Published:** 2022-09-12

**Authors:** Daniela Poli, Emilia Antonucci, Walter Ageno, Paolo Prandoni, Giovanni Barillari, Giuseppina Bitti, Egidio Imbalzano, Eugenio Bucherini, Antonio Chistolini, Vittorio Fregoni, Silvia Galliazzo, Alberto Gandolfo, Elisa Grifoni, Franco Mastroianni, Serena Panarello, Raffaele Pesavento, Simona Pedrini, Girolamo Sala, Pasquale Pignatelli, Paola Preti, Federico Simonetti, Piera Sivera, Adriana Visonà, Sabina Villalta, Rossella Marcucci, Gualtiero Palareti

**Affiliations:** 1 SOD Malattie Aterotrombotiche, Azienda Ospedaliero-Universitaria Careggi, Firenze, Italy; 2 Fondazione Arianna Anticoagulazione, Bologna, Italy; 3 Dipartimento di Medicina e Chirurgia, Università dell'Insubria, Varese, Italy; 4 Medicina Trasfusionale ASU FC Udine; 5 Medicina Interna Ospedale Civile di Fermo, Fermo, Italy; 6 A.O.U. G. Martino, Università di Messina, Messina, Italy; 7 SS Az.le di Angiologia AUSL Romagna, Faenza (RA), Italy; 8 Dipartimento di Medicina Traslazionale e di Precisione, Sapienza Università di Roma, Roma, Italy; 9 U.O.C. Medicina Generale, ASST Valtellina e Alto Lario Ospedale di Sondalo, Italy; 10 UOC Medicina Generale, Ospedale San Valentino, Montebelluna (TV), Italy; 11 SC (UCO) Clinica Medica, ASUGI, Ospedale di Cattinara, Trieste, Italy; 12 Medicina Interna 2, Ospedale San Giuseppe, Empoli (Fi), Italy; 13 UOC Medicina Interna, Covid Unit, EE Ospedale F. Miulli, Acquaviva delle Fonti (Ba), Italy; 14 SC Medicina Interna, Ospedale Galliera Genova, Italy; 15 UO Clinica Medica 3 Azienda Ospedaliero Universitaria Padova, Italy; 16 UO, laboratorio Analisi, Fondazione Poliambulanza Brescia, Italy; 17 UOC Medicina II, Ospedale di Circolo Busto Arsizio (Va), Italy; 18 I Clinica Medica, Medicina Interna Covid e Centro Trombosi Sapienza, Università di Roma, Roma, Italy; 19 Unità di Medicina Interna, Malattie Vascolari e Metaboliche Policlinico San Matteo, Pavia, Italy; 20 UOC Ematologia Aziendale – Ospedale Versilia –Lido di Camaiore (Lucca), Italy; 21 SCDU Ematologia e terapie cellulari, AO Ordine Mauriziano Umberto 1° Torino, Italy; 22 UOC Angiologia, Ospedale San Giacomo Apostolo, Castelfranco Veneto (Treviso), Italy; 23 UOC Medicina Generale, Ospedale San Giacomo Apostolo, Castelfranco Veneto (Treviso), Italy; 24 Department of Experimental and Clinical Medicine, University of Florence, Firenze, Italy

**Keywords:** COVID-19, venous thromboembolism, bleeding, thromboprophylaxis

## Abstract

**Background** Coronavirus disease 2019 (COVID-19) infection causes acute respiratory insufficiency with severe interstitial pneumonia and extrapulmonary complications; in particular, it may predispose to thromboembolic disease. The reported incidence of thromboembolic complications varies from 5 to 30% of cases.

**Aim** We conducted a multicenter, Italian, retrospective, observational study on COVID-19 patients admitted to ordinary wards, to describe the clinical characteristics of patients at admission and bleeding and thrombotic events occurring during the hospital stay.

**Results** The number of hospitalized patients included in the START-COVID-19 Register was 1,135, and the number of hospitalized patients in ordinary wards included in the study was 1,091, with 653 (59.9%) being males and 71 years (interquartile range 59–82 years) being the median age. During the observation, two (0.2%) patients had acute coronary syndrome episodes and one patient (0.1%) had an ischemic stroke; no other arterial thrombotic events were recorded. Fifty-nine patients had symptomatic venous thromboembolism (VTE) (5.4%) events, 18 (30.5%) deep vein thrombosis (DVT), 39 (66.1%) pulmonary embolism (PE), and 2 (3.4%) DVT+PE. Among patients with DVT, eight (44.4%) were isolated distal DVT and two cases were jugular thrombosis. Among patients with PE, seven (17.9%) events were limited to subsegmental arteries. No fatal PE was recorded. Major bleeding events occurred in nine (1.2%) patients and clinically relevant nonmajor bleeding events in nine (1.2%) patients. All bleeding events occurred among patients receiving thromboprophylaxis, more frequently when treated with subtherapeutic or therapeutic dosages.

**Conclusion** Our findings confirm that patients admitted to ordinary wards for COVID-19 infection are at high risk for thromboembolic events. VTE recorded among these patients is mainly isolated PE, suggesting a peculiar characteristic of VTE in these patients.

## Introduction

After the diffusion of the acute respiratory illnesses caused by severe acquired respiratory syndrome coronavirus-2 (SARS-CoV-2) in China, Italy was the first country severely infected by the pandemic, with a widespread diffusion in Northern Italy. 1 The virus causes typical severe interstitial pneumonia, but several extrapulmonary complications have also been reported. In particular, COVID-19 may predispose to both venous and arterial thromboembolic diseases due to excessive inflammation, hypoxia, and immobilization. Moreover, COVID-19-associated coagulopathy has been described that may further increase the thrombotic risk. 2 3 4 In COVID-19 patients, the presence of strong inflammatory markers, endothelial dysfunction, platelet activation, and hypercoagulability has been described. 5 6 The incidence of venous thromboembolism (VTE) was found to be elevated among patients admitted to intensive care units (ICUs), in whom 31% of cases were complicated by thrombotic events, mainly VTE. 3 7 Instead, among patients admitted to ordinary wards, the incidence of thrombotic complications was lower, ranging from 5.8 to 9.2%. 7 8 In particular, the incidence was 6.6% among patients admitted to ordinary wards and systematically treated with thromboprophylaxis. 8

The aim of our study was to evaluate the incidence and the characteristics of the thrombotic complications recorded in patients admitted to ordinary wards and included in the Italian multicenter START-COVID-19 Register in the first wave of the pandemic during the spring of 2020.

## Methods

The START-COVID-19 Register started in May 2020 after the widespread of the COVID-19 pandemic in the frame of the START Register (NCT 02219984). 9 This is a retrospective, observational, nationwide, multicenter register aimed to collect data on the clinical characteristics, laboratory findings, and drugs employed from patients infected by COVID-19, hospitalized in ordinary wards. Patients requiring ICU admission were excluded from the study. The registry was approved by the ethical committee of the Institution of the Coordinating Member (Azienda Ospedaliero-Universitaria, Policlinico S. Orsola-Malpighi, Bologna, Italy) and by all ethical committees of participating centers. All procedures were in accordance with the ethical standards of the institutional and/or national research committee and with the 1964 Helsinki declaration and its later amendments or comparable ethical standards. All patients enrolled in the study read and signed the informed consent. Thirty-one hospitals throughout Italy participated in this data collection. Details of data collection have been reported in a previous study. 10 The registry is aimed to record local practice; therefore, no specific tests or treatments were mandated by the study protocol. All patients underwent nasopharyngeal and oropharyngeal swab sampling on admission, and the presence of COVID-19 infection was detected by the polymerase chain reaction method. A dedicated web-based case report form (e-CRF) obtained with “electronic data capture” system, based on the “Research Electronic Data Capture” online platform (REDCap, produced and distributed by Vanderbilt University and “REDCap Consortium”) 11 was used. The e-CRF collected demographic data, clinical data related to associated diseases, symptoms at admission, and type of treatment. The entity of associated comorbidity was measured using the Charlson comorbidity index. 12

VTE prevention strategies were defined as prophylactic when enoxaparin 4,000 to 6,000 U od, fondaparinux 2.5mg od, nadroparin 2,850 to 3,800 to 5,700 U od, or unfractionated heparin (UFH) 5,000 U bid were used. Treatment was defined subtherapeutic/therapeutic when enoxaparin 4,000 U bid, enoxaparin 6,000 U bid, enoxaparin 8,000 U bid, fondaparinux 5mg to 7.5mg od, or nadroparin 3,800 U bid or 5,600 U bid, or UFH 12.500 U bid were used. Patients were followed-up during hospital stay, the follow-up ended when the patients were discharged, transferred to ICU, or died. The outcome was defined as being favorable when the patient was discharged, and severe when the patient was transferred to ICU or died. Thrombotic and bleeding events occurring during follow-up were recorded. Objective confirmation of thrombotic events was requested. The index event was objectively confirmed by compression ultrasonography, ventilation-perfusion lung scan, or computed tomographic pulmonary angiography. Major bleeding (MB) events were defined according to the International Society of Thrombosis and Haemostasis. 13 Clinically relevant nonmajor bleeding (CRNMB) events were defined as those events that are not major but require any kind of medical intervention. 14

### Statistical Analysis

Descriptive analysis was performed. Continuous variables were expressed as median with interquartile range (IQR) or as mean plus or minus standard deviation. Categorical variables were expressed as frequencies and percentages. Preliminary statistical analysis was performed using Wilcoxon signed-rank test (continuous variables) or Fisher exact test (categorical data). A *p* -value <0.05 was considered statistically significant.

We used the SPSS version 26 software (SPSS Inc, Chicago, IL, United States) for Windows for data processing.

## Results

### Patients

From March 1st and June 30th 2020, 1,135 patients hospitalized for COVID-19 infection were included in the START-COVID-19 Register; 1,091 patients hospitalized in ordinary wards were included in the study, 653 being males (59.9%) with a median age of 71 years (IQR 59–82 years). Characteristics of patients have been previously described. 10 In brief, hypertension was present in 570 (52.2%) patients, the median Charlson's index of the cohort was 3 (range 2–5), and 406 (37.2%) patients had no associated comorbidities. At admission, fever was present in 796 patients (73.0%), dyspnea in 581 (53.3%), and cough in 450 (41.2%).

### Thrombotic Complications

During the observation, two (0.2%) patients had acute coronary syndrome episodes and 1 patients (0.1%) had stroke; no other arterial thrombotic events were recorded. Fifty-nine patients had VTE (5.4%), 18 (30.5%) patients had deep vein thrombosis (DVT), 39 (66.1%) patients had pulmonary embolism (PE), and 2 (3.4%) patients had DVT+PE. Two patients (0.2%) had superficial vein thrombosis ( Table 1 ). Among patients with DVT, eight (44.4%) were isolated distal DVT and two cases were jugular thrombosis. Among patients with PE, seven (17.9%) events were limited to subsegmental arteries. No fatal PE was recorded. PE was diagnosed by CT angiography in 25 cases and by CT scan in 9 cases, and no information was available for 7 cases.

**Table 1:** Thrombotic events occurred during follow-up

	*N* (%)
Venous thromboembolism	59 (5.4)
Deep vein thrombosis	18 (30.5)
Proxymal	10 (55.6)
Isolated distal	8 (44.4)
Jugular vein thrombosis	2 (5.5)
Pulmonary embolism	39 (66.1)
Principal artery	8 (20.5)
Segmentary artery	22 (56.4)
Subsegmentary artery	7 (17.9)
Not available	2 (5.1)
Deep vein thrombosis+Pulmonary embolism	2 (3.4)
Superficial vein thrombosis	2 (0.2)
Stroke	1 (0.1)
Acute coronary syndrome	2 (0.2)

Characteristics of patients with VTE are reported in Table 2 , they presented on the whole a significantly lower number of comorbidities with respect to patients without VTE, even if the median Charlson comorbidity index was similar to patients without VTE. Instead, among VTE patients, median D-dimer levels were significantly higher. The mortality rate was low among patients who had VTE during hospital stay (2 out of 59 patients, 3.4%) with respect to patients without VTE (198 out of 1,032 patients, 19.2%).

**Table 2:** Characteristics of patients with and without venous thromboembolism

	No. of patients with VTE	No. of patients without VTE	*p* -Value
Patients	59	1,032	
Males	37 (62.7)	616 (59.7)	0.7
Median age (IQR), years	69 (58–79)	71 (59–82)	0.6
Body mass index median (IQR)	26.8 (25.2–30.1)	26.0 (24.0–29.1)	0.3
Hypertension	20 (33.9)	550 (53.3)	0.005
Atrial fibrillation	2 (3.4)	81 (8.0)	0.3
Venous thromboembolism	3 (5.1)	33 (3.3)	0.4
Coronary artery disease	3 (5.1)	107 (10.5)	0.3
Heart failure	–	22 (2.1)	–
Peripheral arterial disease	–	16 (1.6)	–
Cerebrovascular disease	2 (3.4)	63 (6.1)	0.5
Neurological disease	7 (11.9)	138 (13.4)	0.8
Chronic obstructive pulmonary disease	4 (6.8)	108 (10.5)	0.5
Rheumatologic disease	–	21 (2.0)	–
Diabetes mellitus	12 (20.3)	178 (17.2)	0.6
Cancer	4 (6.8)	137 (13.3)	0.2
Renal failure (eGFR<30mL/min) a	4 (14.8)	92 (14.7)	1.0
Charlson score, median (IQR)	3 (2–4)	3 (2–5)	0.3
No comorbidities	34 (57.6)	372 (36.3)	0.001
Median D-dimer (mg/dL) (IQR)	1,668 (505–4,385)	779 (407–1,611)	0.03
Prothrombin time ratio, median (range)	1.2 (1.1–1.3)	1.1 (1.0–1.2)	0.1
Thromboprophylaxis	45 (76.3)	724 (70.1)	0.4

Abbreviations: eGFR, estimated glomerular filtration rate; IQR, interquartile range; VTE, venous thromboembolism.

a Available for 653 patients.

At the time of admission, 70.5% of patients of the entire cohort received thromboprophylaxis, mainly with prophylactic doses of enoxaparin. The percentage of treatment was similar among patients who develop and patients who did not develop VTE ( *p* -value=0.4).

### Bleeding Complications

During hospital stay, nine (1.2%) patients had MB events and nine (1.2%) patients CRNMB events. All bleeding events occurred among patients treated with antithrombotic drugs. MBs and CRNMBs occurred more frequently among patients treated with subtherapeutic/therapeutic dosage with respect to patients treated with prophylactic dosage (5 [3.1%] and 6 [3.7%] vs. 4 [0.7%] and 3 [0.5%], respectively).

## Discussion

The principal finding of our study is the confirmation of the rate of thrombotic events reported among patients admitted to ordinary wards for COVID-19 infection. Our results are consistent with those reported by other authors in hospitalized medical patients who are not critically ill, 15 frequency ranging from 5.8 to 9.2%. 7 8 In particular, in an Italian single-center retrospective cohort study, the proportion of COVID-19 patients with VTE was found to be 6.6% in ward patients. The thrombotic events reported are mainly VTE, in particular PE. Our data are in keeping with these findings. In our cohort, 66.1% of VTE events were PE, with a large number of episodes confined to segmental and subsegmental arteries, whereas one-fifth of the episodes involved the main or lobar arteries. None of the events was fatal. Isolated DVT was present in 30% of patients with VTE, and 8 out of 18 (44.4%) DVT cases were limited to the distal veins, this frequency being consistent with that found by other authors 7 16 as was that of PE and overall DVT. 3 8 The mortality rate of VTE patients was impressively lower with respect to that recorded among patients who did not develop VTE during the hospital stay. This unexpected result could be explained by the lower number of comorbidities detected among this group of patients. As a matter of fact, the number of total comorbidities is significantly lower among patients with VTE with respect to patients without, and a trend of lower incidence of diseases associated with higher mortality (coronary artery disease, atrial fibrillation, heart failure, peripheral obstructive arterial disease, cerebrovascular disease, and cancer) is detected among patients without VTE.

The high prevalence of isolated PE (66.1%) found in our cohort is somewhat surprising, as it is in contrast with approximately 20% that was reported in different cohorts of VTE patients without COVID-19 infection. 17 18 We speculate that pulmonary vessel occlusions that are seen in COVID-19 patients are due to the development of local thrombi rather than arising from peripheral veins. 19

A huge number of patients who require hospitalization in the widespread of the pandemic end up limiting the diagnosis of VTE, in particular in those with few or no symptoms. Whenever a systematic leg veins ultrasonography has been performed, a relevant rate of (mainly) asymptomatic episodes has been reported. 20 Therefore, we cannot exclude that an underdiagnosis of VTE in our patients has occurred.

In our cohort, 70.5% of patients received thromboprophylaxis, in the great majority enoxaparin at prophylactic dosage. We confirmed the presence of COVID-19-associated coagulopathy, with elevated median D-dimer levels, higher levels among patients suffering from thrombotic complications. The role of heparin treatment in reducing mortality has been reported by several studies; on the contrary, a clear role in the thromboembolism prevention has not been demonstrated.

We acknowledge the limitations of our study. First, it is a retrospective observational study, and no standardized diagnostic procedure for the detection of thrombotic events was indicated by the study protocol. The severity of patients enrolled, as indicated by the elevated number of patients with fatal outcomes, may have determined underdiagnosis of thrombotic events particularly in the case of pauci- or asymptomatic episodes. The strength of the study lies in the multicentric design and the accuracy and completeness of follow-up for all patients enrolled.

In conclusion, we confirmed that patients admitted to ordinary wards for COVID-19 infection are at high risk for thromboembolic events. VTE recorded among these patients is mainly isolated PE, differently from the incidence of isolated PE commonly observed in the cohorts of VTE patients without COVID-19 infection, suggesting a peculiar characteristic of VTE in these patients.

## List of Participating Centers

Daniela Poli, Rossella Marcucci, SOD Malattie Aterotrombotiche, Azienda Ospedaliero Universitaria-Careggi, Firenze
Walter Ageno, Giovanna Colombo, UOSD Degenza Breve e Internistica, Centro trombosi Ospedale di Circolo, Varese
Chiara Ambaglio, UOSD SIMT Servizio di Immunoematologia e Medicina Trasfusionale, Ospedale di Treviglio – Caravaggio, ASST Bergamo Ovest, Bergamo
Guido Arpaia, U.O. Medicina Interna Carate Brianza ASST-Vimercate
Giovanni Barillari, SOS di Dipartimento “Malattie Emorragiche e Trombotiche, ASUGI, Udine
Giuseppina Bitti, Giuseppe Pio Martino Medicina Interna Ospedale Civile di Fermo
Eugenio Bucherini, Dipartimento Cardiovascolare - Azienda USL di Ravenna, Faenza
Antonio Chistolini, Alessandra Serrao, Dipartimento di Biotecnologie Cellulari ed Ematologia, Azienda Ospedaliero Universitaria Policlinico Umberto I, Roma
Egidio De Gaudenzi, SOC Medicina Interna Ospedale San Biagio – Domodossola
Valeria De Micheli, Ambulatorio Emostasi - Azienda Ospedaliera Di Lecco
Anna Falanga, Teresa Lerede, Luca Barcella, Laura Russo, USC SIMT, Centro Emostasi e Trombosi, Ospedale Papa Giovanni XXIII, Bergamo
Vittorio Fregoni, U.O.C. Medicina Generale, ASST Valtellina e Alto Lario Ospedale di Sondalo.
Silvia Galliazzo, UOC Medicina Generale, Ospedale San Valentino, Montebelluna (TV)
Alberto Gandolfo, Valentina Trapletti, Gianni Biolo, Clinica Medica, Azienda Sanitaria Universitaria Giuliano Isontina (ASU GI) –Ospedale di Cattinara, Trieste
Giorgio Ghigliotti, Clinica Delle Malattie Dell'apparato Cardiovascolare Policlinico San Martino Genova
Elisa Grifoni, Medicina Interna 2, Ospedale San Giuseppe, Empoli (Fi)
Egidio Imbalzano, UOC Medicina Interna, Policlinico di Messina
Gianfranco Lessiani, Unità Angiologica, Dipartimento di Medicina e Geriatria, Ospedale “Villa Serena,” Città Sant'Angelo, Pescara
Giuliana Martini, Sara Merelli, Nicola Portesi Centro Emostasi, Spedali Civili Di Brescia,
Franco Mastroianni, Giovanni Larizza, Covid Unit Subintensiva, EE Ospedale Generale F.Miulli, Acquaviva delle Fonti (Ba)
Serena Panarello, Chiara Fioravanti, SC Medicina Interna, EO Galliera, Genova
Simona Pedrini, Servizio di Laboratorio, Istituto Ospedaliero Fondazione Poliambulanza, Brescia
Raffaele Pesavento, Davide Ceccato, UO Clinica Medica 3 AOU Padova
Pasquale Pignatelli, Daniele Pastori, I Clinica Medica, Medicina Interna Covid e Centro Trombosi
Sapienza, Università di Roma
Paola Preti, Centro Emostasi e Trombosi, IRCCS Fondazione Policlinico S. Matteo, Pavia
Girolamo Sala, Fabrizio Foieni, Michela Provisone, Ospedale di Circolo Busto Arsizio (Va)
Luca Sarti, Antonella Caronna, Paolo Vita, Medicina Interna e Area Critica, Ospedale di Baggiovara (Mo)
Federico Simonetti, Ilaria Bertaggia, UOC Ematologia Aziendale – Ospedale Versilia –Lido di Camaiore (Lucca)
Piera Sivera, Carmen Fava, S.C.D.U. Ematologia e terapie cellulari, AO Ordine Mauriziano Umberto 1° Torino
Adriana Visonà, Beniamino Zalunardo, UOC Angiologia, Ospedale San Giacomo Apostolo, Castelfranco Veneto (Treviso)
Sabina Villalta, UOC Medicina Generale, Ospedale San Giacomo Apostolo, Castelfranco Veneto (Treviso)
